# Dietary regulation of PI3K/*AKT*/GSK-3β pathway in Alzheimer’s disease

**DOI:** 10.1186/alzrt265

**Published:** 2014-06-20

**Authors:** Yasuko Kitagishi, Atsuko Nakanishi, Yasunori Ogura, Satoru Matsuda

**Affiliations:** 1 Department of Food Science and Nutrition, Nara Women’s University, Kita-Uoya Nishimachi, Nara, 630-8506, Japan

## Abstract

Alzheimer’s disease (AD) is characterized by the formation of senile plaques and neurofibrillary tangles composed of phosphorylated Tau. Several findings suggest that correcting signal dysregulation for Tau phosphorylation in AD may offer a potential therapeutic approach. The PI3K/*AKT*/GSK-3β pathway has been shown to play a pivotal role in neuroprotection, enhancing cell survival by stimulating cell proliferation and inhibiting apoptosis. This pathway appears to be crucial in AD because it promotes protein hyper-phosphorylation in Tau. Understanding those regulations may provide a better efficacy of new therapeutic approaches. In this review, we summarize advances in the involvement of the PI3K/*AKT*/GSK-3β pathways in cell signaling of neuronal cells. We also review recent studies on the features of several diets and the signaling pathway involved in AD.

## Introduction

Alzheimer’s disease (AD) is neuro-pathologically characterized by the damage of neurons and synapses as well as the formation of senile plaques from amyloids and neurofibrillary tangles (NFTs) composed of hyper-phosphorylated Tau, which is the most prevalent cause of age-related dementia [1-3]. Soluble amyloid species, including oligomers, may alter hippocampal synaptic plasticity and impair memory [4,5]. The hyper-phosphorylated Tau is the principal component of helical filaments in intracellular NFTs. Amyloid-beta (Aβ) deposition occurs prior to the accumulation of the hyper-phosphorylated Tau in the AD brain. Soluble Aβ oligomers isolated from the brain extract of patients with AD accelerate Tau hyper-phosphorylation [6,7]. In spite of the observations representing the pathophysiological roles of soluble Aβ species in AD pathogenesis, how Aβ induces the hyper-phosphorylation of Tau in AD brains remains an unanswered question. Although research efforts have provided insights into the biology of AD, the underlying routes mediating the progressive decline in cognitive function are still poorly understood. The precise molecular events that control the death of neuronal cells with Aβ are unclear.

The characterization of full-length Tau has shown that the Tau protein can undergo many transitional conformations, and each of the conformations may represent a potentially toxic object. Accumulation of the misfolded Tau intermediates in the human brain causes tauopathies, the most common form of AD [8]. The PI3K/*AKT*/GSK-3β pathway appears to be crucial for AD because it promotes protein hyper-phosphorylation in Tau. In particular, glycogen synthase kinase-3β (GSK-3β) plays a key role in the neuronal response to stress by phosphorylating and compromising the transcriptional activity of the cAMP response element binding, which regulates the transcription of the brain-derived neurotrophic factor (BDNF) and other neuropeptides that are important in the regulation of long-term memory and in the maintenance of synaptic plasticity, thereby contributing to the pathology of neuronal degeneration [9,10]. Furthermore, GSK-3β is probably the most documented kinase implicated in the abnormal hyper-phosphorylation of Tau protein.

Several potential preventive factors against AD have been suggested by epidemiological research, including modifiable lifestyle factors such as diet [11]. Researchers have demonstrated that dietary choices can play an important role in the neuroprotection of AD. Because many factors in life influence brain function, several interventions might be promising in the prevention of brain dysfunction in AD [12]. The main objective of this article is to review the studies linking potential protective factors to pathogenesis of AD, focusing particularly on the roles of the PI3K/*AKT*/GSK-3β pathway.

## Tau phosphorylation involved in the PI3K/*AKT*/GSK-3β signaling pathway

**Figure 1:**
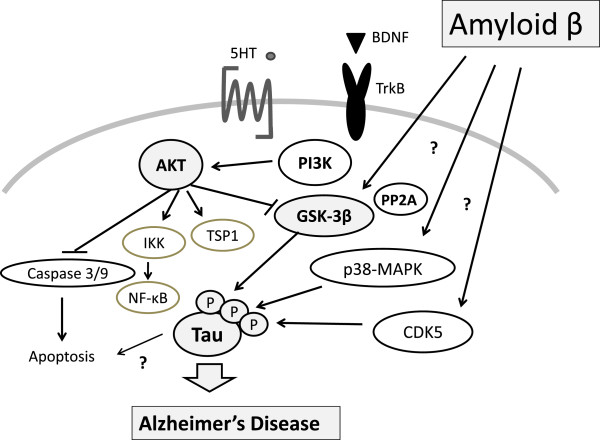
**Schematic representation of PI3K/AKT/GSK-3β signaling pathway in neuronal cells of Alzheimer’s disease.** Examples of molecules known to act on the regulatory pathways are shown. Note that some critical pathways have been omitted for clarity. 5-HT, 5-hydroxytryptamine, serotonin; BDNF, brain-derived neurotrophic factor; CDK5, cyclin-dependent kinase 5; GSK-3β, glycogen synthase kinase-3β; IKK, IκB kinase; NF-κB, nuclear factor-kappa-B; p38-MAPK, p38 mitogen-activated protein kinase; PI3K, phosphoinositide-3 kinase; PP2A, protein phosphatase 2A; TrkB, tropomyosin-receptor-kinase B; TSP1, thrombospondin-1.

Tau proteins are essential in assembly as well as maintenance of the structural integrity of microtubules [13-15]. However, Tau is abnormally hyper-phosphorylated and aggregated in AD. Aβ induces neuronal death and hyper-phosphorylation of the Tau protein (Figure 1), which is the main event responsible for NFT formation in AD brains [16]. Previous studies have demonstrated differential roles of Tau phosphorylation at several phosphorylation sites. It is apparent that Tau phosphorylation at various sites influences Tau activity. A consequence of Tau hyper-phosphorylation in AD is a reduction in its ability to bind microtubules, a destabilization of microtubule network, NFT formation, and ultimately neuronal death [17]. Disordered microtubule-associated Tau proteins are susceptible to aggregation. Aberrant Tau hyper-phosphorylation is implicated in neurodegeneration in AD. Several kinases and phosphatases have been identified to regulate the phosphorylation of Tau. Tau has been found to be phosphorylated at over 30 serine/threonine residues in the brains of patients with AD, and approximately one half of these are canonical sites for proline-directed protein kinases, including GSK-3β, cyclin-dependent kinase 5, and p38 mitogen-activated protein kinase (p38-MAPK) [18,19]. These kinases are involved primarily in Tau hyper-phosphorylation [20,21]. Tau phosphorylation at the proline-rich region, which is located upstream of the microtubule-binding domains, inhibits its microtubule assembly activity moderately and promotes its self-aggregation slightly [22]. Tau phosphorylation at the C-terminal tail region increases its activity and promotes its self-aggregation markedly [23]. Tau phosphorylation at both of these regions plus the microtubule-binding region nearly diminishes its activity and disrupts microtubules. Abnormally hyper-phosphorylated Tau disengages from microtubules and then increased cytosolic concentrations of unbound Tau occur, resulting in NFT [24,25]. Hyper-phosphorylated Tau is the major component of the paired helical filaments that accumulate in degenerating neurons in AD and other neurodegenerative diseases.

**Figure 2:**
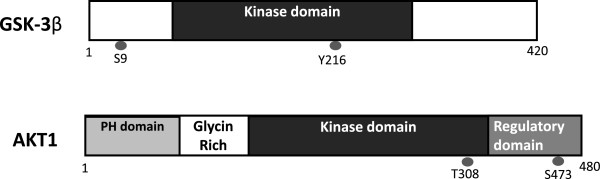
**Schematic structures of human AKT1 and GSK-3β protein.** The predicted consensual domain structures for each protein are depicted. The functionally important sites including the sites of protein phosphorylation are shown. Note that the sizes of protein are modified for clarity. GSK-3β, glycogen synthase kinase-3β; PH, pleck-strin-homology.

*AKT* inhibition may contribute to the decrease of Tau phosphorylation at Thr212 and Ser214 because these two sites are substrates of *AKT*[26,27]. *AKT* phosphorylation is catalyzed mainly by phosphoinositide-3 kinase (PI3K)-phosphoinositide-dependent protein kinase-1 (PDK1). Phosphatidylinositol 3,4,5-triphosphate (PIP3) is the major second messenger of the PI3K pathway that mediates receptor tyrosine kinase signaling to the survival kinase *AKT*. Increased levels of PIP3 at the membrane cause pleck-strin-homology (PH) domain-containing proteins such as *AKT* and PDK1 to colocalize, resulting in the kinase-mediated phosphorylation and activation [28]. Thus, activated PI3K induces the activation of *AKT*, which phosphorylates various biological substrates, including GSK-3β. PI3K/*AKT* signaling pathway dysfunction causes GSK-3β activity increase and leads to Tau hyper-phosphorylation, the main component of NFT [29]. GSK-3β has been shown to phosphorylate Tau in intact cells on multiple sites, some of which are aberrant in the abnormally hyper-phosphorylated Tau protein, a critical event in AD pathogenesis. Accordingly, a PI3K inhibitor (such as wortmannin) increases Tau hyper-phosphorylation [30,31]. In addition, a study has suggested that PI3K/*AKT* signaling is attenuated in the brains of patients with AD [32]. Generally, under an *AKT*-activated process, complete activation requires Ser473, so levels of Ser473 phosphorylation represent the degree of *AKT* phosphorylation. GSK-3β is rendered inactive when it is phosphorylated at Ser9 by activated *AKT*[33]. Schematic structures of human *AKT*1 and GSK-3β protein are shown in Figure 2. The activated *AKT* phosphorylates target proteins involved in cell survival, cell cycling, angiogenesis, and metabolism for neuroprotection (Figure 1). In other words, selective downregulation of the *AKT* concurrent with elevated GSK-3β activity may be linked to brain dysfunctional pathogenesis. It has been shown that *AKT* activation may play a therapeutic role in neurodegenerative diseases [34]. *AKT* is an important regulator of cell survival and apoptosis. GSK-3β is ubiquitously active and is a critical effector of PI3K/*AKT* cellular signaling. Thus, several cellular processes such as cell metabolism, cell death, and survival depend on GSK-3β [35,36].

Protein phosphatase 2A (PP2A) is the major protein phosphatase in the brain that dephosphorylates Tau at several phosphorylation sites, thereby stopping the ability of Tau to inhibit microtubule assembly and to self-assemble into helical filaments and NFTs [37]. PP2A activity has been shown to be decreased in AD brains [38]. GSK-3β antagonized by PP2A regulates Tau phosphorylation at many sites. PP2A has been reported to dephosphorylate GSK-3β at Ser-9 [39]. In contrast, activation of GSK-3β can inhibit PP2A. The mammalian target of rapamycin (mTOR) also regulates the activity of PP2A, and the inhibition of the mTOR activates the PP2A [40]. When the PI3K/*AKT*/mTOR signaling is affected, a regulatory interaction between PP2A and GSK-3β, which ensures the steady Tau phosphorylation, changes [41]. This equilibrium seems to be established by the regulatory coupling of mTOR by a dephosphorylation of GSK-3β mediated by PP2A. Another regulatory factor for PP2A activity is peptidyl-prolyl *cis-trans*-isomerase 1 (Pin1), a phosphorylation-dependent prolyl cis/trans isomerase. This isomerase has been shown to stimulate the dephosphorylation of Tau by PP2A [42]. In Pin1 knockout mice, Tau is hyper-phosphorylated [43]. Pin1 also regulates Tau stability and phosphorylation dynamics [44]. Pin1 promotes amyloid precursor protein (APP) turnover by inhibiting GSK-3β activity [45]. Pin1 changes are a constant feature of AD pathology and could serve as a biomarker of the onset or spread of AD neuropathology [46]. Overexpressing Pin1 or preventing Pin1 inhibition might be a new approach to block tauopathy.

## Some dietary agents may contribute to the regulation of the PI3K/*AKT*/GSK-3β pathway

Several medicinal herbs and foods may regulate the activity of the PI3K/*AKT*/GSK-3β pathway. *Achyranthes bidentata* Blume is a commonly prescribed Chinese medicinal herb. The *Achyranthes bidentata* polypeptides might exert their protective effects against neuronal apoptosis through modulation of the PI3K/*AKT*/GSK-3β pathway [47]. *Phyllanthus niruri* also activates the PI3K/*AKT* pathway. Puerarin, the main isoflavone glycoside found in the Chinese herb, functions through activation of PI3K/*AKT* signaling [48]. GSK-3β has been shown to be significantly inhibited by the treatment of berberine extracted from *Coptis chinensis* Franch, a Chinese medicinal herb [49]. In addition, dietary long-chain omega-3 polyunsaturated fatty acids may affect membrane-associated signaling proteins such as *AKT*[50]. Unfortunately, however, we have found no clinical application in the literature of these dietary agents against AD treatment. Lithium increases cell survival by inducing BDNF and thereby stimulating activity in anti-apoptotic pathways, including the PI3K/*AKT* pathway, which has been proposed to function as a neuroprotective agent that prevents neuronal apoptosis [51-53]. Lithium is also known to both directly and indirectly regulate GSK-3β through activation of the PI3K/*AKT* and MAPK signaling pathways in neurons. This regulation of GSK-3β is believed to be one of the main mechanisms by which lithium effects its neuroprotective role, as regulation of the kinase leads to downstream expression of anti-apoptotic and cell survival genes. Lithium has been involved in GSK-3β-related reduction in glutamate activity involved in neuronal survival [54,55].

Leptin signaling also induces the activation of the ubiquitous nutrient-sensitive PI3K/*AKT*/mTOR pathway. Studies have demonstrated that leptin induces the activation of *AKT* via phosphorylation of *AKT* at Ser473. As a consequence, *AKT* activation ensues upon leptin signaling, which results in inhibition of GSK-3β through phosphorylation at Ser9 residue [56]. Obviously, leptin also activates the serine/threonine kinase mTOR in the hypothalamus through the PI3K/*AKT* pathway [57]. The mTOR is an evolutionary conserved kinase that controls translation of several mRNA transcripts involved in cell growth and cell proliferation. Pathways activated by leptin terminate in the phosphorylation of the Tau kinase GSK-3β at Ser9 residue, leading to the inhibition of its kinase activity. Therefore, leptin induced activation of *AKT*, p38-MAPK, and Tau kinase GSK-3β signal transduction pathways [58]. Branched-chain amino acid leucine activates the intracellular mTOR signaling pathway [59], which is critical for initiating the protein translation process [60]. The dried root of *Anthriscus sylvestris* has been used in traditional drugs for the treatment of various diseases. Anthricin, which is a natural product isolated from *A. sylvestris*, is an inhibitor of mTOR [61].

## Potential therapeutic approach of the dietary agents for the neuroprotection in Alzheimer’s disease via the inhibition of Tau-phosphorylation

The culinary herb sage (*Salvia officinalis*) may be effective for patients with mild to moderate AD [62]. Rosmarinic acid, a major ingredient of sage, reduced a number of events induced by Aβ, including Tau protein hyper-phosphorylation. These data show the neuroprotective effect of sage against Aβ-induced toxicity, which could validate the traditional use of this spice in the treatment of AD. The clinical relevance has been emphasized by the observation that patients did not experience any adverse effect while taking sage. Pharmacological activities of sage relevant to AD include antioxidant activity, anti-inflammatory effects, and cholinesterase inhibition and particularly its reputation for aiding memory [63]. However, despite these promising clinical observations, the precise mechanism for this herb remains elusive. Rosmarinic acid is also a major ingredient of lemon balm (*Melissa officinalis*), a plant that has shown promising signs of therapeutic activity in patients with AD. Main activities of rosmarinic acid include anti-inflammatory, antioxidant, antibacterial, and antiviral properties [64].

Curcumin, a component of turmeric, can also improve structure and plasticity of synapse and enhance learning and memory abilities [65]. Interestingly, curcumin enhances synaptic plasticity and cognitive function in rats [66], suggesting that curcumin may represent a potent therapeutic agent which exerts multiple beneficial effects. It is suggested that the neuroprotection of curcumin might be mediated via the PI3K/*AKT* signaling pathway [67]. Genistein potentiates the anti-cancer effects of gemcitabine in human osteosarcoma via the downregulation of the *AKT* pathway [68]. Long-term treatment with lithium has been shown to modify performance deficits in transgenic mice expressing human APP by inhibiting GSK-3β [69]. This improvement in performance was associated with an increased *AKT* activation and an improved GSK-3β phenotype in neurons.

**Figure 3:**
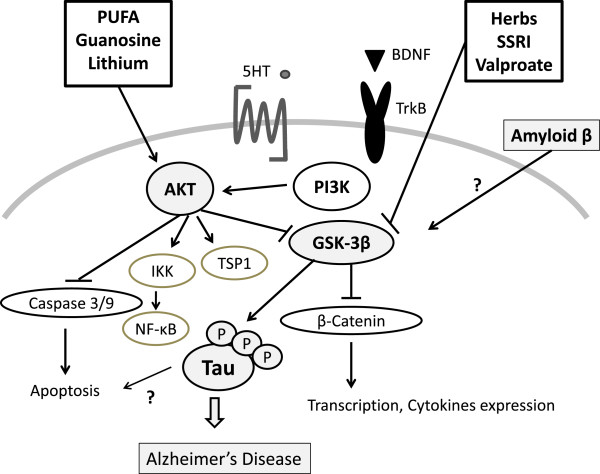
**Potential molecular targets based on the predominant PI3K/AKT/GSK-3β pathway.** The targets suggest that certain diets and medicines may contribute to neuro-protection via modulating the function of AKT and GSK-3β in Alzheimer’s disease. Note that some critical events have been omitted for clarity. 5-HT, 5-hydroxytryptamine, serotonin; BDNF, brain-derived neurotrophic factor; GSK-3β, glycogen synthase kinase-3β; IKK, IκB kinase; NF-κB, nuclear factor-kappa-B; PI3K, phosphoinositide-3 kinase; PUFA, polyunsaturated fatty acid; SSRI, selective serotonin reuptake inhibitor; TrkB, tropomyosin-receptor-kinase B; TSP1, thrombospondin-1.

Ghrelin amends the effect of high glucose on decreased neuronal Tau hyper-phosphorylation, which has a positive correlation with the degree of cognitive dysfunction. Leptin also increases synaptogenesis and aids in memory formation in the hippocampus and is purported to be a cognitive enhancer. Epidemiological studies have implicated decreased leptin levels in the pathogenesis of AD. The leptin levels are inversely related to the risk of developing dementia of the AD type [70]. Evidence suggests that leptin facilitates spatial learning and memory [71,72] and also increases neurogenesis in mice. Leptin decreases hyper-phosphorylation of Tau by the activation of the signal transduction pathway coupled to leptin receptors, which eventually mitigates Tau phosphorylation. It was shown that leptin is more potent than insulin at mitigating Tau phosphorylation [73,74]. Thus, neuroprotecton in AD could be performed by certain diets and medicines involved in the PI3K/*AKT*/GSK-3β pathway (Figure 3).

## Perspective

Clarification of the molecular mechanisms by which extracellular Aβ induces the hyper-phosphorylation of Tau in the pathogenic pathway of AD is essential, and Tau may be responsive to pharmacological intervention before neurodegeneration occurs. Tau is abnormally hyper-phosphorylated at multiple phosphorylation sites, and thus loses its ability to bind to microtubules and is part of pathological lesions characterizing tauopathies in AD. Experimental studies have shown that Aβ induces Tau hyper-phosphorylation in a number of cell types. Incompetent activation of *AKT*/GSK-3β signaling may be relevant to Aβ-induced Tau phosphorylation. A coordinated regulation of PP2A and GSK-3β seems to ensure balanced Tau phosphorylation. This may help in preventing severe changes in Tau phosphorylation. It is conceivable that different pathways function in concert with Tau phosphorylation in the AD brain. Understanding the effects of Aβ on the hyper-phosphorylation of Tau may provide a new therapeutic approach. However, the pathology of AD is complex and may involve several different biochemical pathways, including defective Aβ protein metabolism and inflammatory, oxidative, and hormonal pathways. Consequently, these pathways are all potential targets for AD treatment and prevention strategies.

Diet usually consists of complex combinations of lipids or nutrients that might act synergistically or antagonistically. One of the pleiotropic properties of these foods could explain their disease protective potentials, which could be mediated through modulation of the PI3K/*AKT*/GSK-3β pathway. Although the precise mechanisms have not yet been clarified, correcting PI3K/*AKT*/GSK-3β signal dysregulation by certain dietary agents in the central nervous system may be a potential therapeutic approach for some patients with AD. Further mechanistic studies are needed in order to understand the precise molecular mechanisms and to determine whether an adequate dietary intake is related to improved brain function and to determine the role it plays regarding the preservation of brain health. Long-term clinical studies are mandatory to elucidate the effect of treatment in the management of AD.

## Abbreviations

AD: Alzheimer’s disease; APP: Amyloid precursor protein; Aβ: Amyloid-beta; BDNF: Brain-derived neurotrophic factor; GSK-3β: Glycogen synthase kinase-3β; mTOR: Mammalian target of rapamycin; NFT: Neurofibrillary tangle; p38-MAPK: p38 mitogen-activated protein kinase; PDK1: PI3K-phosphoinositide-dependent protein kinase-1; PI3K: Phosphoinositide-3 kinase; Pin1: Peptidyl-prolyl *cis-trans*-isomerase 1; PIP3: Phosphatidylinositol 3,4,5-triphosphate; PP2A: Protein phosphatase 2A.

## Competing interests

The authors declare that they have no competing interests.
